# Comparative neuroprotective effects of Cerebrolysin, dexamethasone, and ascorbic acid on sciatic nerve injury model: Behavioral and histopathological study

**DOI:** 10.3389/fnana.2023.1090738

**Published:** 2023-02-01

**Authors:** Heba M. Elhessy, Ola A. Habotta, Mamdouh Eldesoqui, Wael M. Elsaed, Mona F. M. Soliman, Haitham M. Sewilam, Y. H. Elhassan, Nermeen H. Lashine

**Affiliations:** ^1^Department of Anatomy and Embryology, Faculty of Medicine, Mansoura University, Mansoura, Egypt; ^2^Department of Forensic Medicine and Toxicology, Faculty of Veterinary Medicine, Mansoura University, Mansoura, Egypt; ^3^Department of Basic Medical Sciences, College of Medicine, Almaarefa University, Riyadh, Saudi Arabia; ^4^Department of Histology and Cell Biology, Faculty of Medicine, Mansoura University, Mansoura, Egypt; ^5^Department of Histology and Cell Biology, Faculty of Medicine, Helwan University, Helwan, Egypt; ^6^Department of Anatomy, College of Medicine, Taibah University, Medina, Saudi Arabia

**Keywords:** sciatic nerve, dexamethasone, Cerebrolysin, apoptosis, ascorbic acid, sciatic function index (SFI), peripheral nerve injury (PNI)

## Abstract

**Background:**

The majority of the suggested experimental modalities for peripheral nerve injury (PNI) result in varying degrees of recovery in animal models; however, there are not many reliable clinical pharmacological treatment models available. To alleviate PNI complications, research on approaches to accelerate peripheral nerve regeneration is encouraged. Cerebrolysin, dexamethasone, and ascorbic acid (vitamin C) drug models were selected in our study because of their reported curative effects of different mechanisms of action.

**Methodology:**

A total of 40 adult male albino rats were used in this study. Sciatic nerve crush injury was induced in 32 rats, which were divided equally into four groups (model, Cerebrolysin, dexamethasone, and vitamin C groups) and compared to the sham group (*n* = 8). The sciatic nerve sensory and motor function regeneration after crushing together with gastrocnemius muscle histopathological changes were evaluated by the sciatic function index, the hot plate test, gastrocnemius muscle mass ratio, and immune expression of S100 and apoptosis cascade (BAX, BCL2, and BAX/BCL2 ratio).

**Results:**

Significant improvement of the behavioral status and histopathological assessment scores occurred after the use of Cerebrolysin (as a neurotrophic factor), dexamethasone (as an anti-inflammatory), and vitamin C (as an antioxidant). Despite these seemingly concomitant, robust behavioral and pathological changes, vitamin C appeared to have the best results among the three main outcome measures. There was a positive correlation between motor and sensory improvement and also between behavioral and histopathological changes, boosting the effectiveness, and implication of the sciatic function index as a mirror for changes occurring on the tissue level.

**Conclusion:**

Vitamin C is a promising therapeutic in the treatment of PNI. The sciatic function index (SFI) test is a reliable accurate method for assessing sciatic nerve integrity after both partial disruption and regrowth.

## 1. Introduction

Both central and peripheral nervous systems are primarily made up of nerve cells which have limited renewal capability. The peripheral nervous system is more vulnerable to physical harm than the central nervous system because it lacks physical defenses such as the skull and vertebrae (Stevens, 2016). Unlike the central nervous system, the peripheral nervous system is gifted to repair and regenerate after injury, but this capability is functionally limited and slow with subsequent vulnerability to permanent disabilities. Overall the process of peripheral nerve recovery is far from being satisfactory (Minegishi et al., 2022).

Peripheral nerve injuries (PNIs) are estimated to occur in 2.8% of multiple traumas and expand to 5% if nerve roots and/or plexus injuries are included. PNIs may be classified as mechanical, including traumatic injuries and iatrogenic lesions, and neuropathic, which can have different etiologies, such as diabetes mellitus, chemotherapy, chemicals, or alcohol (Bota and Fodor, 2019). Posttraumatic PNIs occur as a result of either secondary to complete transection of the nerve or partial nerve injury as a result of compression or inflammation (Lopes et al., 2022). Unlike complete transection, which is managed surgically, partial injury is usually managed medically (Ferreira et al., 2005).

In restorative medicine, posttraumatic PNIs repair is a major challenge since it can result in temporary or permanent sensory and/or motor harms, which can lead to eventual financial and social concerns (Jiang et al., 2016). Therapeutic agents, surgical approaches, and immune system modulators have been proposed to enhance nerve regeneration (Xavier et al., 2012; de Souza et al., 2021). However, none of the tried policies restored the preinjury motor and sensory function (Geuna et al., 2009).

A variety of therapeutic modalities have been adopted by physicians to achieve recovery in animal models, yet attaining fully functional recovery is still a challenge to be addressed (Hussain et al., 2020). Many antioxidants (vitamin E, ubiquinone, and L-carnitine) (Karsidag et al., 2012; Azizi et al., 2014; Moradi et al., 2014), anti-inflammatories (dexamethasone and acetylsalicylic acid) (Subbanna et al., 2007; Feng and Yuan, 2015), or other substances (diphosphocholine, rapamycin, and tetanus toxin) (Gunay et al., 2014; Ding et al., 2015; Emril et al., 2016) have been tried. However, few effective clinical drug treatments are available. It is necessary to continue investigating how to determine the ideal therapy for PNI by establishing standardized injury models and proper regenerative assessment methods (Lopes et al., 2022).

Cerebrolysin (hydrolysated cerebroproteins) is a neuropeptide preparation that mimics the action of endogenous neurotrophic factor containing free amino acids and was first used in 1973 (Zhovnir et al., 1973). It is widely used in degenerative and acquired central nervous system insults such as dementia, stroke, and traumatic injuries. Its action was attributed to its ability to ameliorate the effects of oxidative stress, reduce apoptosis, and promote neuronal growth besides having the ability to penetrate the blood–brain barrier (Sharma et al., 2010; Masliah and Diez-Tejedor, 2012). Several studies showed that Cerebrolysin also ameliorates peripheral nerve injuries (Hamed, 2011; Dong et al., 2016).

Dexamethasone is a well-known glucocorticoid anti-inflammatory that is frequently used to heal neural inflammation following injury. The preventive effects of topically applied dexamethasone were previously shown in a rat study of peripheral nerve crush (Sun et al., 2012). In addition, local dexamethasone treatment enhanced peripheral nerve regeneration and reinnervation in a transected sciatic nerve rat model (Mohammadi et al., 2013). This action was explained by the efficacy of dexamethasone’s local administration to inhibit CD3-positive cell infiltration with the upregulation of GAP-43 expression (Feng and Yuan, 2015).

Ascorbic acid, or vitamin C, is a dietary element that is needed for several biological functions (Granger and Eck, 2018). It is necessary for the growth and physiological processes of nerve tissue (May, 2012) and has also therapeutic effects in many neurodegenerative diseases (Kocot et al., 2017; Moretti et al., 2017). Research has proven its ability in alleviating spinal cord injury and enhancing functional nervous recovery (Geyh et al., 2012; Guo et al., 2018). Ascorbic acid appears to have antinociceptive effects on PNI animals, according to the available data (Saffarpour and Nasirinezhad, 2017; Li et al., 2019).

In this study, we evaluated the effectiveness of Cerebrolysin, dexamethasone, and vitamin C on PNI considering the recovery on both motor and sensory levels. The results were correlated with coincident behavioral and histopathological changes. The pertinency for the use of the sciatic function index (SFI) test as a single non-invasive tool reflecting the histopathological state of the sciatic nerve was also targeted.

## 2. Materials and methods

### 2.1. Ethical statement

Rat care and all experimental procedures including surgery, behavior tests, and tissue collection were carried out following the Research Ethics Committee, Faculty of Veterinary Medicine, Mansoura University code No: R/138. All efforts were made to minimize the number of animals used and their suffering.

### 2.2. Experimental animals

In this investigation, 40 mature male albino rats (weighing 200–250 g) were used. The rats were kept in a pathogen-free environment with a 12-h light/dark cycle and unrestricted access to food and water pellets.

### 2.3. Surgical procedure and grouping

Rats were randomly split into five equal groups after 2 weeks of acclimatization. The sciatic nerve crush technique was chosen to elicit acute blunt sciatic nerve injury because it produces a lesion that is comparable to those exhibited by patients with PNIs and offers a standard direct trauma. We followed previously approved guidelines implementing this well-established model in order to reduce animal-to-animal variation (Bélanger et al., 2011; Moore et al., 2012; Renno et al., 2013). The exact same forceps [Micro mosquito forceps 12.5 cm (S) (500451) (09F S/N) (World Precision Instruments, Inc., Fl, USA)] were used in each surgery by the same researcher at the same location at the precise location of the sciatic nerve (10 mm distal to the sciatic notch) for 60 s. In short, the rats were anesthetized with an intraperitoneal injection of 10% chloral hydrate (3 ml/kg body weight) (Feng and Yuan, 2015). The left leg was shaved and washed with an antiseptic solution before positioning for surgery. The left sciatic nerve was exposed through a gluteal muscle-splitting incision at the mid-thigh level. Thirty-two rats underwent surgery, and complete crush was confirmed by the presence of a translucent band across the nerve, while the sciatic nerve was exposed but not crushed in eight rats (the sham group). The incision was then closed in layers (muscle and skin) with absorbable sutures (Feng and Yuan, 2015).

All rats were maintained under identical conventional laboratory conditions and received standard postoperative care as previously described in Ramli et al. (2017). Each rat received 1 ml of Lactated Ringer’s solution subcutaneously and 0.4 mg/kg of Baytril intramuscularly. The incision was treated with 10 mg/g of chloramphenicol and 5 mg/g of hydrocortisone acetate ointment.

Animals were grouped as follows: The sham group (*n* = 8), in which, despite the sciatic nerve being exposed, it was not crushed. The crush-lesioned rats (*n* = 32) were randomly distributed to reduce group differences as much as feasible. In the model group (*n* = 8), no medication was given after crushing the rats till the time of scarification. In the Cerebrolysin group (*n* = 8), rats were injected intraperitoneally with 8.98 mL/kg of Cerebrolysin (purchased from EVER Neuro Pharma Cat. N 1097018) for 10 days (Dong et al., 2016). In the dexamethasone group (*n* = 8), dexamethasone was topically administered by intramuscular injection around the site of nerve injury on both legs to reduce the side effects of glucocorticoids administered systemically (2 mg/kg purchased from the AMRIYA pharm Product Code: 13283). Injections were given once a day for 10 days in accordance with the clinical protocol and rat weight (Feng and Yuan, 2015). In the vitamin C group (*n* = 8), rats received 400 mg/kg ascorbic acid solution *via* intragastric administration by a customized probe through which the solution could be pushed forward into the stomach on the first day, and then 200 mg/kg every day for 10 days. Vitamin C (purchased from Sigma-Aldrish, Merck KGaA, Darmstadt, Germany, Germany Code N A4403) was prepared in a suspension with saline at a concentration of 13.33 mg/ml (Li et al., 2019). The control samples were considered by taking the right legs of all groups as a control to the left operated legs.

### 2.4. Assessment of recovery (motor and sensory)

The motor and sensory functional recovery of the injured hindlimb of rats receiving sciatic nerve crush injury was detected 13 days after injury by the sciatic function index test and hot plate, which are widely used in sciatic nerve crush injury models. Each mouse was allowed at least a 2-h break between testing sessions. All behavior tests were formally performed 13 days after injury, but the rats were trained for 2 days before the final tests. Behavioral tests were performed and scored by trained operators blind to experimental conditions.

#### 2.4.1. Motor function assessment

**FIGURE 1:**
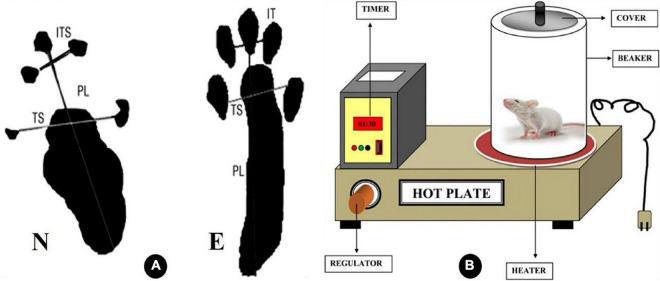
Diagrammatic illustration of the variables considered in calculating the sciatic function index (SFI) in panel **(A)**. IT intermediate toes, PL print length, and TS toes spread, as N is the normal leg and E is the experimental leg A (Barbosa et al., 2010). **(B)** Explanatory diagram of hot plate analgesiometer (Patel et al., 2016).

Motor function recovery was appraised using the SFI as previously described (Jahromi et al., 2020). Rats were permitted to pass along a wooden passage (8.2 cm width and 42 cm long) toward the end of a dark enclosure to collect at least five footprints after their hind paw legs had been dipped in blue ink. They left behind several identifiable footprints that were lined up on paper. The following parameters were measured in animal imprints on the experimental (E) and control normal (N) sides. Toe spread (TS), the distance between the first and fifth toes, and intermediary toe (IT) spread, the distance between the second and fourth toes, were used to determine the print length (PL), toe spread (TS), and intermediary toe (IT) spread, respectively. Using the formula provided by Bain and colleagues, SFI was determined in rats (Bain et al., 1989): *SFI* = −38.3 (*EPL − NPL*)/*NPL* + 109.5 (*ETS − NTS*)/*NTS* + 13.3 (*EIT − NIT*)/*NIT* − 8.8, as shown in Figure 1A. The test results were assessed as follows: An SFI value of −100 indicates a serious complete malfunction, whereas a score of 0 denotes a healthy nerve.

#### 2.4.2. Sensory function assessment

Sensory function recovery was appraised by the hot plate test (HPT) (Mulder and Pritchett, 2004). The hot plate test is used as a model of pain sensitivity and assessment of sensory function as it measures a complex behavior that requires neurological processing within the brain. First rats were brought into the testing room, and a period of acclimatization is permitted (30–60 min). The surface of the hot plate is cleaned with 70% ethanol before use. Rats were positioned on a heated surface that is maintained between 52 and 55°C, and to prevent rats from moving off the platform, a transparent plastic cylinder is placed around the animal as shown in Figure 1B. A few seconds later, rats will lift and lick a paw in response to heat. The animal is then immediately removed from the apparatus. The dependent variable in this test is the time, or latency, to lick the paw, and it is manually recorded using a stopwatch. Some rats may jump or vocalize, and these responses may also be used in place of paw licking. A cutoff time, 30 s, was established to minimize the risk of permanent tissue injury from prolonged exposure to the heated surface.

### 2.5. Samples collections

After the completion of the behavioral tests on the 13th day, rats were deeply anesthetized with an intraperitoneal injection of 50 mg/kg tiletamine plus zolazepam and 15 mg/kg xylazine hydrochloride.

#### 2.5.1. Blood samples

Blood serum specimens were collected and placed into tubes containing ethylenediaminetetraacetic acid (EDTA). Centrifugation at 3,000 × *g* for 10 min at room temperature was performed to obtain serum samples and then stored at −80°C till the time of analysis.

#### 2.5.2. Sciatic nerve specimen

Each rat was nailed supinely to a dissecting board. Using scissors, a very small longitudinal incision (approximately 5.0 mm) was done along the lateral aspect of the thigh to reveal the whole length of the sciatic nerve, which is visible as a thick whitish cord in the thigh region. The skin was retracted, and the posterior thigh muscles, including the hamstring muscles, were then moved. Forceps were used to carefully elevate the nerve, and the proximal and distal ends of the nerve were removed to obtain the intermediate section. A part of the sciatic nerve was placed in an Eppendorf tube for biochemical analysis and stored at −80°C. The other part was embedded in paraffin after being treated with 4% paraformaldehyde for 24 h for histopathological assessment.

#### 2.5.3. Gastrocnemius muscle specimen

Gastrocnemius muscles were isolated and fixed with 4% paraformaldehyde for 24 h and then embedded in paraffin.

### 2.6. Biochemical evaluation (serum and tissue oxidative stress markers)

Malondialdehyde (MDA) concentration (thiobarbituric acid reactive substances) was measured as described in Kiasalari et al. (2017). Superoxide dismutase (SOD) activity was assayed in accordance with Türedi et al. (2018). Catalase (CAT) was obtained using the method described in Türedi et al. (2018).

### 2.7. Histological assessment of sciatic nerve tissue for morphological myelin alterations

Sectional thinness Leica semithin microtome was used to slice sections 1 μm thick. The sciatic nerves were then stained with Luxol fast blue (LFB) as previously described in Sajadi et al. (2020) for myelin sheath evaluations (color intensity, myelin thickness, and nerve fiber diameter).

### 2.8. Histological assessment of gastrocnemius muscle

The tissue sections (5 μm) were subjected to hematoxylin and eosin (H&E) staining (Lee et al., 2020). We assessed the area occupied by muscle fibers by using the image analysis software program as previously described in Li et al. (2019).

### 2.9. Immunohistochemical study of the sciatic nerve

For the immunohistochemical analysis, paraffin-embedded sections were dewaxed and hydrated, and endogenous peroxidase activity was inhibited with 0.03% hydrogen peroxide (H2O2). Exposing the antigenic site by boiling the sections in 0.01 M citrate buffer for 20 min. The samples were treated with 5% normal goat serum in phosphate buffered saline (PBS) for 20 min to prevent non-specific binding. S100 (rabbit polyclonal anti-S100 protein, 1:100 dilution; Abcam code N 34686), CD3 (cluster of differentiation rabbit monoclonal antibody 1:150, Abcam, code N ab16669), BCL2 (B-cell lymphoma 2 rabbit monoclonal antibody, 1:50 dilution; Abcam, code N ab194583), and BAX (BCL2-associated X Protein rabbit monoclonal antibody 1:250 dilution; code N ab32503) were incubated on the sections at 4°C for an overnight period. Then, as a secondary antibody, the horseradish peroxidase (HRP)-conjugated goat antirabbit polyclonal antibody was used, and 3,3′-diaminobenzidine (DAB) (Abcam cat. N 64264) was used as the chromogenic substrate solution as previously described in Luo et al. (2021).

### 2.10. Image analysis

Five randomly selected sections of each animal in each group were studied under a 40X objective (area: 0.071 mm2). For legitimate comparisons, the homogeneity of section thickness was taken into account. Light microscope (Olympus model BX53, Tokyo, Japan) images were captured using a digital camera (ToupCam type BX53, Japan) linked to a computer. The LFB color intensity and immune positive reaction of S100, BAX, and BCL2 were analyzed using Image-J (Fiji is just). For analyzing LFB sections, we used the tool color histogram measuring the color intensity (Jahromi et al., 2020), taking into account that S100 immune positive reaction was presented in Schwan cells forming the myelin sheet and that BCL2 and Bax immune-positive reaction was observed in both cytoplasm and nuclei of the nerve cells. For the analysis of the immune positive reaction, we used the color deconvolution icon from the image and then measured the area percentage of brown color using the threshold tool as described by Heddleston et al. (2021). The area fraction of BAX immunoexpression was multiplied by the area fraction of BCl2 immunoexpression to obtain the BAX/BCL2 ratio as described in Sedaghat (2021).

### 2.11. Statistical analysis

Data were presented as the mean ± SD. IBM SPSS Statistics 26 (SPSS Corporation, Tokyo, Japan) was used to conduct the statistical analysis. A test of normality was applied to verify normality distribution. One-way repeated measures ANOVA and a *post hoc* comparison test were used to evaluate all data. Correlations between SFI and hot plate test, SFI and BAX/BCL2, SFI and SR100, and LFB intensity versus muscle mass ratio were plotted, and Spearman’s correlation coefficients were labeled. The results were considered significant at a *P*-value of < 0.05.

## 3. Results

None of the experimental rats experienced postoperative complications (e.g., autotomy and dehiscence). No animal died during or after surgery.

### 3.1. Assessment of functional recovery

#### 3.1.1. Motor function

**FIGURE 2:**
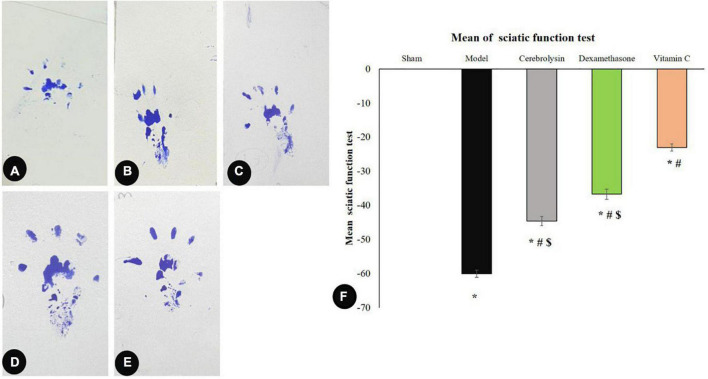
Sciatic function index data: **(A–E)** representing rats’ footprints; sham **(A)**, injury **(B)**, Cerebrolysin **(C)**, dexamethasone **(D)**, and vitamin C **(E)**. **(F)** Histogram illustrating that after damage, the sciatic functional index (SFI) significantly decreased in comparison to the sham group. The SFI value was significantly higher in the vitamin C group versus the Cerebrolysin and dexamethasone groups. The difference between the Cerebrolysin and dexamethasone groups was not significant. *Significance as compared to control, ^#^significance as compared to the model, and ^$^significance as compared to Vit C group.

As shown in Figure 2, the SFI value was significantly decreased (*P* < 0.05) in the model group and correlated with the sham group. Cerebrolysin, dexamethasone, and vitamin C were also found to significantly improve SFI (*P* < 0.05) and thus motor activity. We also noticed that vitamin C enhanced SFI more significantly than Cerebrolysin and dexamethasone groups. There was no visible difference between Cerebrolysin and dexamethasone groups.

#### 3.1.2. Sensory function

**FIGURE 3:**
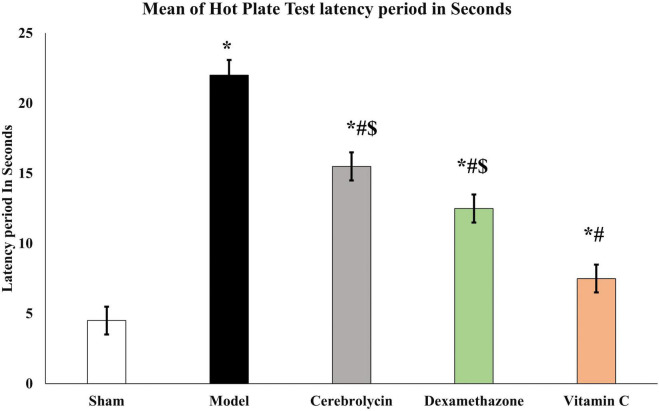
Hot plate test histogram representing the withdrawal latency period of a hot plate test. *Significance as compared to control, ^#^significance as compared to the model, and ^$^significance as compared to Vit C group.

The recovery of sensory function was evaluated using the hot plate test. Sciatic nerve injury caused significant prolongation (*P* < 0.05) of the latency period as compared to the sham group which was interpreted as a sluggish sensory response. In comparison to the model group, Cerebrolysin, dexamethasone, and vitamin C treatment shortened the latency period significantly (*P* < 0.05). The vitamin c group significantly decreases the latency period as compared to both Cerebrolysin and dexamethasone groups as shown in Figure 3.

### 3.2. Biochemical evaluation for oxidative stress marker

In the model rats, a significant elevation (*P* < 0.05) in the MDA with a significant decrease (*P* < 0.05) in the SOD and CAT levels was decreased compared with the sham group. Treatment with Cerebrolysin, dexamethasone, and vitamin C group significantly decreased the level of MDA (*P* < 0.05) and significantly increased SOD and CAT levels (*P* < 0.05) compared to the model group (Table 1). It was observed that vitamin C has a more enhancing impact as antioxidant therapy.

**TABLE 1:** Mean level of the malondialdehyde (MDA), superoxide dismutase (SOD), and catalase (CAT) in serum and sciatic nerve tissue of rats ± SD in all groups and *N* = 8 for each group.

	MDA level		SOD level		CAT level	
	Mean ± SD		Mean ± SD		Mean ± SD	
	Serum (μ /ml)	Tissue (n\mg)	Serum (μ /ml)	Tissue (μ /mg)	Serum (μ /ml)	Tissue (kμ /mg)
Sham	0.185 ± 0.024	4.393 ± 1.050	7.380 ± 1.006	1.600 ± 0.20	23.956 ± 0.172	3.135 ± 0.415
Model	0.377 ± 0.031	9.013 ± 1.975	1.396 ± 0.236	0.377 ± 0.070	12.648 ± 0.781	1.092 ± 0.179
Cerebrolysin	0.240 ± 0.210*^#$^	8.200 ± 1.313*^$^	2.686 ± 0.427*^#$^	0.663 ± 0.151*^#$^	15.567 ± 0.662*^#$^	1.776 ± 0.294*^#$^
Dexamethasone	0.280 ± 0.220*^#$^	6.433 ± 1.424^#^	4.176 ± 0.296*^#$^	0.932 ± 0.101*^#$^	18.231 ± 651*^#$^	2.405 ± 0.2644*^#$^
Vitamin C	0.330 ± 0.202*^#^	5.117 ± 1.340*^#^	6.272 ± 0.322*^#^	1.273 ± 0.066*^#^	20.136 ± 673*^#^	2.812 ± 0.254*^#^

*Significance as compared to control, ^#^significance as compared to model, and ^$^significance as compared to Vit C group. *P*-value < 0.05.

### 3.3. Histopathological evaluation

As there was no difference in the histopathological data of both the control (right leg of all the experimental rats) and the sham groups, they were treated as a single group and given the term control.

#### 3.3.1. Morphometric observations of the sciatic nerve

**FIGURE 4:**
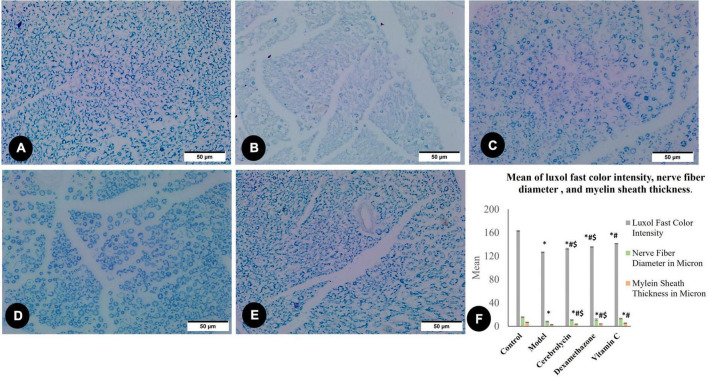
Luxol fast blue staining myelin sheet in the control group **(A)**, the model group **(B)**, the Cerebrolysin group **(C)**, the dexamethasone group **(D)**, and the vitamin C group **(E)**. **(F)** Histogram illustrates a comparison of the Luxol fast color intensity, myelin sheath thickness in microns, and nerve fiber diameter in microns between different groups. *Significance as compared to control, ^#^significance as compared to the model, and ^$^significance as compared to Vit C group.

The existence of nerve myelin sheaths was observed and evaluated using light microscopic slices of the sciatic nerve stained with LFB (Figures 4A–E). Morphometric results concerning Luxol’s fast color intensity, myelin sheath thickness, and nerve fiber diameter are presented in Figure 4F. Figure 4A represents a photomicrograph of the control rat, and it shows the normal distribution of axon size, number, and myelin thickness. The control group shows the highest color intensity, myelin sheath thickness, and nerve fiber diameter. Figure 4B depicts an erratic axon with a thin myelin sheath. The color intensity, myelin thickness, and nerve fiber diameter significantly (*P* < 0.05) decreased in the model group correlated with the control group. There was an ameliorative effect of the Cerebrolysin (Figure 4C), dexamethasone (Figure 4D), and vitamin C (Figure 4E) groups on the myelin and nerve fiber, and this was shown as a significant increase (*P* < 0.05) in LFB color intensity, myelin thickness, and nerve fiber diameter when compared to the control group (Figure 4F).

#### 3.3.2. Histological assessment of gastrocnemius muscle

**FIGURE 5:**
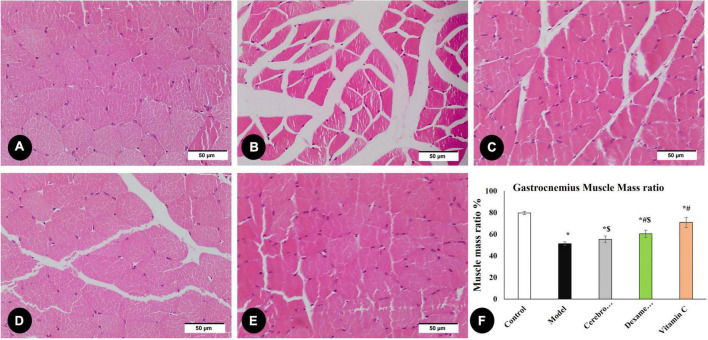
H&E staining of the gastrocnemius muscle in the control group **(A)**, the model group **(B)**, the Cerebrolysin group **(C)**, dexamethasone group **(D)**, and vitamin C **(E)**. **(F)** Histogram illustrates a comparison of area percentage of gastrocnemius muscle fibers between different groups. *Significance as compared to control, ^#^significance as compared to the model, and ^$^significance as compared to Vit C group.

By measuring the area percentage of gastrocnemius muscle fibers, Figure 5 illustrates the impact of various therapies on muscle atrophy after sciatic nerve damage. The area percentage of gastrocnemius muscle fibers significantly decreased (*P* < 0.05) in the model group as compared to the control group. Cerebrolysin, dexamethasone, and vitamin C significantly increase the ratio (*P* < 0.05) as compared to the model group.

#### 3.3.3. Evaluation of nerve regeneration (S100 area percentage)

**FIGURE 6:**
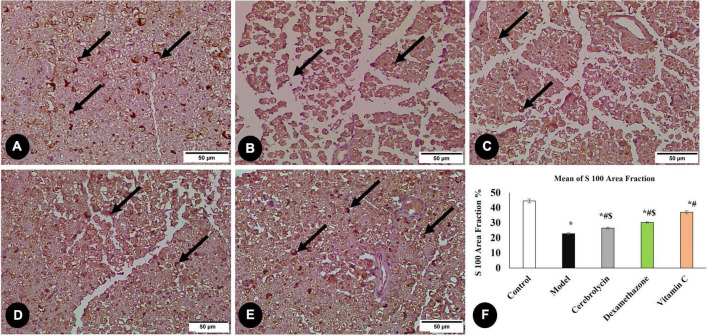
Immunohistochemical analysis of S100 marker in the control group **(A)**, the model group **(B)**, the Cerebrolysin group **(C)**, the dexamethasone group **(D)**, and vitamin C **(E)**. Arrows point to positively stained cells. **(F)** Comparison of the expression between different groups. S100 expression was significantly decreased in the model group as compared to the control group. The Cerebrolysin group, dexamethasone group, and vitamin C cause a significant increase in S100 expression correlated with the model group. There was no significant difference between Cerebrolysin and dexamethasone. *Significance as compared to control, ^#^significance as compared to the model, and ^$^significance as compared to Vit C group.

A critical stage in the regeneration and functional repair of damaged peripheral nerves is remyelination. Using S100, a mature Schwan Cell marker, researchers have been able to track SC proliferation and myelin sheath development (Ma et al., 2019). Figure 6 illustrates S100 immune expression in different studied groups. S100 immune expression in the model group was significantly decreased as compared to the control group. In the vitamin C, Cerebrolysin, and dexamethasone-treated groups, S100 expression was significantly increased in comparison with the model group. The vitamin C-treated group showed a significant increase in S100 expression as compared to Cerebrolysin and dexamethasone-treated groups.

#### 3.3.4. Evaluation of inflammatory marker CD3 (CD3 cell counting)

**FIGURE 7:**
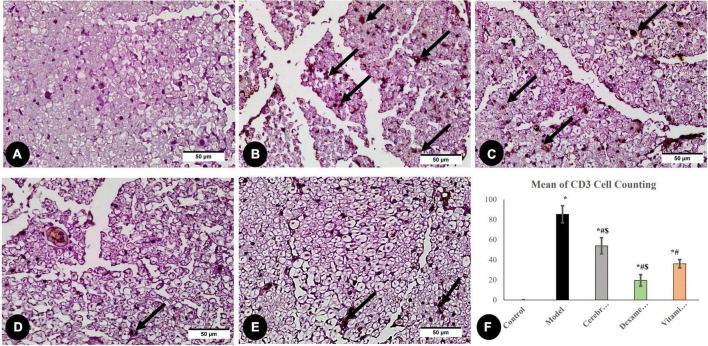
Immunohistochemical analysis of CD3 marker: in the control group **(A)**, the model group **(B)**, the Cerebrolysin group **(C)**, the dexamethasone group **(D)**, and the vitamin C group **(E)**. Arrows point to positively stained cells. **(F)** Comparison of the expression between different groups. CD3 cell counting was significantly increased in the model group as compared to the control group. The Cerebrolysin group, dexamethasone group, and vitamin C cause a significant decrease in CD3 cell counting correlated with the model group. *Significance as compared to control, ^#^significance as compared to model, and ^$^significance as compared to Vit C group.

Large numbers of CD3-positive cells were discovered in the model group denoting that sciatic nerve damage prompted a T-cell recruitment response. As shown in Figure 7, CD3-positive cells decreased significantly in all three treated groups compared to the model group.

#### 3.3.5. Assessment of apoptosis cascade (BAX, BCL2, and BAX/BCL2 ratio)

**FIGURE 8:**
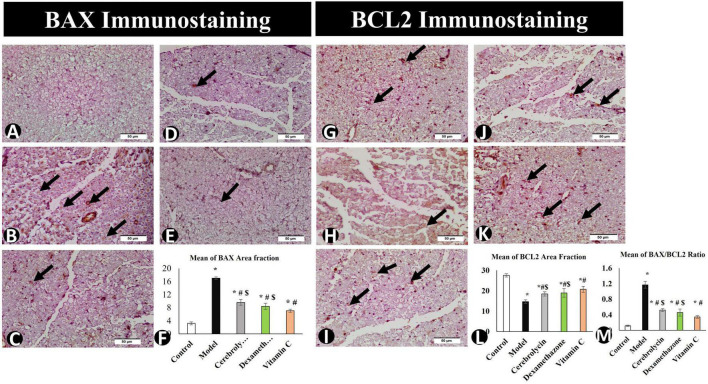
BAX immunostaining in the control group **(A)**, the model group **(B)**, the Cerebrolysin group **(C)**, the dexamethasone group **(D)**, and the vitamin C group **(E)**. **(F)** Comparison of the expression between different groups. BAX expression was significantly decreased in the model group as compared to the control group. The Cerebrolysin group, dexamethasone group, and vitamin C cause a significant increase in BAX expression correlated with the model group. There was no significant difference between Cerebrolysin and dexamethasone. BCL2 immunostaining in the control group **(G)**, the model group **(H)**, the Cerebrolysin group **(I)**, the dexamethasone group **(J)**, and vitamin C **(K)**. Arrows point to positively stained cells. **(L)** Comparison of the expression between different groups. BCL2 expression was significantly decreased in the model group as compared to the control group. The Cerebrolysin group, the dexamethasone group, and the vitamin C group cause a significant increase in BCL2 expression correlated with the model group. **(M)** Bax/Bcl2 ratio increased significantly (*P* < 0.005) in the model as compared to the control group. Dexamethasone, Cerebrolysin, and vitamin C significantly reduced the Bax/Bcl2 ratio compared to the model group (*P* < 0.005). *Significance as compared to control, ^#^significance as compared to model, and ^$^significance as compared to Vit C group.

After the interpretation of the BAX and BCL2 stained sections, BAX expression was significantly higher in the model group than in the control group, whereas BCL2 expression was significantly decreased. The vitamin C, Cerebrolysin, and dexamethasone-treated groups showed a significant decrease in BAX expression as compared to the model group and a significant increase in Bcl2 expression as compared to the model group. The vitamin C-treated group showed a significant increase in BCL2 expression and a significant decrease in BAX expression as compared to Cerebrolysin and dexamethasone-treated groups (Figure 8).

To determine the BAX/BCL2 ratio, immune expression data for BAX and BCL2 were analyzed (Figure 8M) to emphasize the ability of Cerebrolysin, dexamethasone, and vitamin C to adapt or overcome sciatic nerve injury. This study found that Cerebrolysin, dexamethasone, and vitamin C significantly decreased the ratio emphasizing its efficacy in alleviating SNI.

### 3.4. Correlation study

**FIGURE 9:**
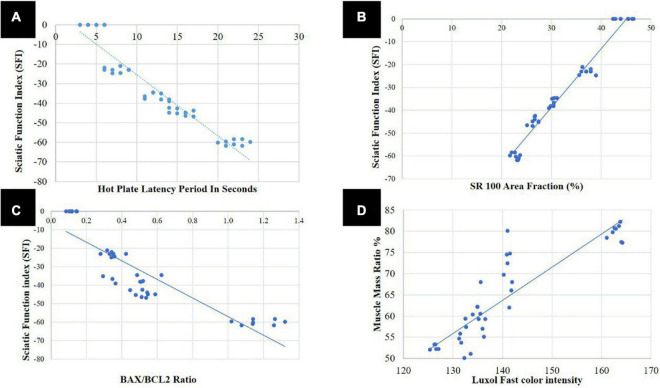
Correlation analysis; **(A)** between sciatic function index (SFI) and hot plate latency period in seconds, **(B)** between SFI and S100 area fraction, **(C)** between SFI and BAX/BCL2 ratio, and **(D)** between area percentage of gastrocnemius muscle fibers and Luxol fast color intensity ratio.

As shown in Figure 9A, using Spearman s correlation, there was a highly significant negative correlation of strong association between the sciatic function test and latency period of the hot plate test (*r* = 0.912; *p* < 0.001), indicating that both motor and sensory recovery are strongly correlated. There was a highly significant positive correlation of strong association between SFI and SR100 area percentage (*r* = 0.970; *p* < 0.001) (Figure 9B) and between SFI and BAX/BCL2 ratio (*r* = 0.787; *p* < 0.001) (Figure 9C). Denoting that sensory and motor function improvement is correlated with histopathological improvement and SFI can be used as a single non-invasive reliable parameter for improvement after sciatic nerve crush.

Area percentage of gastrocnemius muscle fibers was also strongly correlated with LFB color intensity (*r* = 0.805; *p* < 0.001) (Figure 9D). Denoting that muscle is affected either negatively or positively in SNI and with the use of different therapeutics, respectively, the same as myelin sheath of the nerve.

## 4. Discussion

In medical practice, posttraumatic PNI repair is a challenge as it is usually incomplete and ends with sensory and motor impairments (Jiang et al., 2016). Experimentally, partial sciatic nerve crush is a reliable experimental model of PNI (An et al., 2022). The induced degeneration/regeneration process is dynamic and the cascade of events after injury starts with both ischemic and inflammatory activities (Hsu et al., 2016). This process of injury induces the generation of oxidative stress markers and enhances lipid peroxidation (Naik et al., 2006; Senoglu et al., 2009). Nerve cells defend themselves by the expression of phase-II detoxification and antioxidant enzymes to combat the induced toxic damage (Johnson et al., 2008). Accordingly, antioxidants, anti-inflammatory, and neurotrophic factors are expected to enhance regeneration and repair (Qiu et al., 2014; Hsu et al., 2016). Although many drugs have been tried in PNI research, the optimal level of recovery is still far away.

We studied in parallel the behavioral and histopathological changes following PNI to detect the correlation and the reliability of behavioral tests as an indicator of the nerve state.

The motor functional recovery after PNI was evaluated by SFI to assess the restoration of regeneration of nerves and foot muscle (Monte-Raso et al., 2008). Our results evidenced improved SFI with Cerebrolysin and dexamethasone with an advantage of vitamin C. Similar results were previously reported (Feng and Yuan, 2015; Dong et al., 2016; Li et al., 2019).

The hot plate test detected sensory functional recovery of variable degrees in the studied groups (Goecks et al., 2012). The data derived from the motor and sensory functional tests illustrated that nerve integrity is improved by treatment with three drugs. The three drug-treated groups recovered significantly from the controls, and vitamin C also had the best results.

Histopathologically, LFB staining revealed an abnormal distribution of the myelin sheaths, decreased color density, decreased myelin sheath thickness, and nerve fiber diameter after PNI. These changes were overturned under the effect of three drugs. S100-labeled Schwann cell immune increased in comparison with the control indicating improved nerve regeneration (Magill et al., 2007). The vitamin C-treated group showed the highest immune area fraction expression percentage. S100 activity diminished in the Schwann cells following nerve injury represents differentiation into a more immature phenotype that does not forcefully express S100 (Feng et al., 2000). The progressive increased S100 expression after the crush injury marks Schwann cell maturation.

At the same time, the area percentage of gastrocnemius muscle fibers was restored considerably. It is well-established that denervation caused by PNI results in amyotrophy in the target muscles. When regenerating axons reach the muscles and reinnervate them, this process is reversed, which explains why the three medications improved the area percentage of gastrocnemius muscle fibers (Pak et al., 2016).

The neuroregenerative efficacy of Cerebrolysin was attributed mainly to its structure, as it is composed of low-molecular-weight peptides and amino acids with neuroprotective properties, such as insulin-like growth factors 1 and 2 and glial cell-derived neurotrophic factor (Kim et al., 2019). It is particularly alluring to link dexamethasone’s actions at the location of injury with its immunosuppressive and possible neurotrophic effects resulting in a diminished generation of inflammatory cells and inflammatory mediators such as upregulation of GAP-43 expression and prevention of CD3-positive cell infiltration (Feng and Yuan, 2015). However, the superiority of vitamin C can be attributed to its higher antioxidant impact and also coincides with previous reports that it has a role in myelin formation, *in vitro* (Li et al., 2019), and *in vivo* (Shokouhi et al., 2005). These proteins were all consistently and noticeably elevated after being exposed to vitamin C. According to our research, vitamin C alters the behavior of Schwann cells, which improves nerve regeneration (Shokouhi et al., 2005).

Apoptosis data on BAX and BCL2 immune expression were used to accurately estimate the cells’ sensitivity to cell death by achieving the BAX/BCL2 ratio (Amin et al., 2014). The BCL2 family and BAX are known to be crucial mediators of the apoptotic process (Danial and Korsmeyer, 2004). This process is promoted by BAX and other subgroups that are similar to BAX, while the BCL2 family acts as an apoptotic suppressor (Youle and Strasser, 2008). After chronic sciatic nerve constriction in rats, a functional imbalance between proapoptotic BAX and antiapoptotic BCL2 has been identified (Maione et al., 2002). The decrease in the BAX/BCL2 ratio emphasizes the antiapoptotic effect of Cerebrolysin (Lu et al., 2022), the antiapoptotic effect of local dexamethasone in agreement with previous studies (Hoang et al., 2009; Lee et al., 2015), and the antiapoptotic effect of vitamin C to adapt or overcome Sciatic nerve injury (Sourour et al., 2012; Gong et al., 2022). Similarly, the antiapoptotic effect of vitamin C was superior to Cerebrolysin and dexamethasone, which may be referred to as its ability in neutralizing free radicals and ameliorating oxidative stress-mediated cellular apoptosis and cell death.

The different investigation methodologies in the follow-up of PNI increase the need for dependable effective evaluation tests. To confirm our tests’ results, statistical correlation formulae proved a significant negative correlation between the sciatic nerve function test and the latency period. There was a significant positive correlation between SFI and SR100 area percentage and among SFI and BAX/BCL2 ratio. Muscle mass ratio was also strongly correlated with LFB color intensity in the histopathological study. These formulae correlate sensory and motor function with histopathological improvement. Accordingly, SFI can be used as a single non-invasive reliable parameter for improvement after sciatic nerve crush.

## 5. Conclusion

This study proved the efficacy of three different drug groups: neurotrophic, anti-inflammatory, and antioxidants in the management of PNI. Vitamin C as an example of antioxidants showed the best results. The correlation between sensory and motor functions’ improvements with histopathological improvement can be proven by SFI as a single non-invasive test for follow-up.

## 6. Recommendation

Further studies involving a combination of different drug groups are recommended.

## Data availability statement

The raw data supporting the conclusions of this article will be made available by the authors, without undue reservation upon request.

## Ethics statement

The animal study was reviewed and approved by the Research Ethics Committee, Faculty of Veterinary Medicine, Mansoura University Code No: R/138.

## Author contributions

HE: conception, design of the research, data collection, data analysis and interpretation, drafting the manuscript, critical revision, and final approval. OH: methodology. WE: conception, share in drafting the manuscript, critical revision, and final approval. ME: revision, additional work, and modification needed by the reviewers. MS and HS: revision and final approval. YE: data collection, analysis, and revision. NL: data analysis and interpretation and final revision. All authors contributed to the article and approved the submitted version.
